# A QoS-Adaptive Interference Alignment Technique for In-Band Full-Duplex Multi-Antenna Cellular Networks

**DOI:** 10.3390/s22239417

**Published:** 2022-12-02

**Authors:** Ki-Hun Lee, Gyudong Park, Bang Chul Jung

**Affiliations:** 1 Department of Electronics Engineering, Chungnam National University, Daejeon 34134, Republic of Korea; 2 Command and Control Systems PMO, Agency for Defense Development, Daejeon 34186, Republic of Korea

**Keywords:** cross-link interference (CLI), degrees-of-freedom (DoF), in-band full-duplex (IBFD) cellular networks, interference alignment (IA)

## Abstract

In this paper, we propose a novel interference alignment (IA) technique for an in-band full-duplex (IBFD) multiple-input multiple-output (MIMO) cellular network where a base station (BS) and user equipment (UE) are equipped with multiple antennas, and the local channel state information (CSI) is available at all nodes. Considering a practical IBFD MIMO cellular network, it is assumed that only the BS operates with full-duplex (FD) communication while UE operate in half-duplex (HD) mode. These IBFD networks introduce a new type of interference called cross-link interference (CLI), in which uplink UE affects downlink UE. The proposed IA technique consists of two symmetric IA schemes according to the number of antennas in the uplink and downlink UE, and both schemes effectively mitigate CLI in the IBFD MIMO network. It is worth noting that both IA schemes are adaptively applicable according to the network’s quality-of-service (QoS) requirements, such as uplink and downlink traffic demands. Furthermore, we theoretically characterize and prove the achievable sum-degrees-of-freedom (DoF) of the proposed IA technique. Simulation results show that the proposed IA technique significantly improves the sum rate performance compared to conventional HD communications (multi-user MIMO) while achieving the same achievable DoF as the interference-free IBFD MIMO network.

## 1. Introduction

Next-generation cellular networks require significantly higher data rates and spectral efficiencies than modern commercial mobile communication systems to accommodate the surging traffic demands. Recently, in-band full-duplex (IBFD) communication has emerged as one of the promising technologies to fulfill these quality-of-service (QoS) requirements in sixth-generation (6G) wireless communication systems [1,2]. Compared to half-duplex (HD) operation deployed in current cellular networks, full-duplex (FD), especially IBFD, theoretically doubles the system capacity and spectral efficiency by allowing overlap between transmission and reception of signals over the same time and frequency radio resources [3,4,5]. For many years it has been considered impractical because of the high power difference between the high-power self-interference from one’s own transmit antenna to its receiver antenna and the low-power desired signal received from the corresponding transmitter. Enormous efforts have gone into investigations to realize the IBFD operation for wireless communication systems, and as a result, significant progress has been made. Specifically, various techniques to suppress the self-interference have been designed and proposed in the literature, including three major techniques such as antenna placement [6], analog-circuit-domain cancellation [7], and digital-domain cancellation [8], which gradually alleviate the self-interference from radio-frequency (RF) stage to baseband stage. For more details, see [9,10,11,12] and the references therein. From now on, FD means IBFD unless otherwise stated.

In practical cellular networks, the FD operation may only be supported by the base station (BS) because it is nontrivial to implement on the user equipment (UE) due to the extra hardware burden [13,14]. In this case, the FD BS can simultaneously communicate with downlink and uplink UE through the same radio resources and accommodate more UE if there are more antennas at the BS than the UE. On the other hand, the aforementioned FD techniques can effectively suppress self-interference in the BS, but a new source of interference, called cross-link interference (CLI), is introduced between UE because the signal transmitted from the uplink UE to the BS is also received at the downlink UE which is expected to receive the signal only from the BS. There is the potential to double spectral efficiency even if the FD operation is applied only to the BS; however, the expected benefits could be spoiled if the CLI is not adequately managed.

Several interference management techniques against the CLI have been recently studied within the context of exploiting interference alignment (IA) and a degrees-of-freedom (DoF) optimal approach [15]. Interference is a traditional major impairment factor for the QoS, such as data rate and reliability in multi-user cellular networks, and IA is a prominently investigated technique for interference management [15,16,17,18,19,20]. Briefly, the basic idea of IA is to project (align) unintended signals (interference) into a signal subspace orthogonal to the desired signal direction through cooperative pre- and post-coding at the transmitter and receiver, respectively. As summarized in the literature [18], IA techniques have been vigorously investigated, considering dimensions, network topologies, applications, and fundamental aspects: feasibility condition, performance metrics, and channel state information (CSI). Opportunistic IA (OIA) has also been extensively investigated as one of the most practical realizations of IA techniques for cellular networks [21,22,23,24,25,26]. More recently, an iterative IA scheme for a cognitive radio (CR) network has been investigated [27]. Meanwhile, in [28], an ergodic IA technique has been proposed to alleviate the CLI in an IBFD cellular network with multi-antenna FD BS and single-antenna FD UE. Asymptotic IA techniques have been proposed to deal with the CLI for the case of a multi-antenna FD BS communicating with single-antenna HD UE in [29,30], and for the case of an FD BS communicating with either HD or FD UE [14]. They have shown remarkable results for IA in IBFD networks, but there are some limitations in that all nodes require global (full) CSI, and multiple antennas are not considered for the UE.

To make IA more implementable, different types of IA have been studied. In [31], a one-shot linear IA technique has been proposed to mitigate the CLI in an IBFD multiple-input multiple-output (MIMO) network with FD BS and HD UE, but it still requires global CSI on both sides. The authors of [32] have investigated blind IA techniques that require no CSI or partial CSI at the transmitter, but it is limited to the case of reconfigurable antennas. In [13], a linear IA technique has been proposed to alleviate the CLI between a multi-antenna FD BS and HD UE when imperfect CSI is available at the transmitter. In [33], a blind IA technique has been investigated for high mobility IBFD vehicular networks with no CSI at the transmitter. To narrow down the signaling overhead due to the CSI feedback [18,34], the authors of [35] have proposed a distributed IA technique in which each node only utilizes local (own) CSI, but it is designed for the case of a BS communicating with UE in dynamic time-division duplex mode.

In this paper, we propose a novel IA technique operating under local CSI to mitigate the CLI in general IBFD MIMO networks with FD BS and HD UE. In summary, our main contributions to this paper are as follows:

- To completely eliminate the CLI in an IBFD MIMO network, it is necessary to take the appropriate number of equipped antennas for each node, also called the *feasibility condition* of IA [31]. We define IBFD MIMO cellular network scenarios to which IA techniques can be applied practically.
- We present two IA schemes based on the distributed manner that can alleviate the signaling overhead caused by CSI sharing between nodes [18,34]. Moreover, both IA schemes exploit linear beamforming techniques without an iterative algorithm.
- We theoretically characterize and prove the achievable DoF of the proposed IA schemes and show that they achieve the same DoF as the interference-free IBFD MIMO network.

It is also worth noting that the proposed two IA schemes can be adaptively applied according to uplink and downlink QoS requirements, such as traffic demands.

The remainder of this paper is organized as follows. In Section 2, we describe the system and signal models of the considered IBFD MIMO network. In Section 3, we design two IA schemes and mathematically characterize the achievable DoF. In Section 4, simulation results are presented. Conclusions are drawn in Section 5.

## 2. System Model and Preliminaries

We consider an in-band full-duplex (IBFD) MIMO cellular network consisting of a base station (BS) with 2M antennas, $N_{d}$ downlink user equipment (UE) with $L_{d}$ antennas each, and $N_{u}$ uplink UE with $L_{u}$ antennas each. Here, we assume that only the BS has an FD capability since self-interference cancellation may not be feasible in UE, and that 2M antennas in the BS are separated by *M* each for transmission and reception (of course, the proposed IA technique can be straightforwardly extended even when the number of transmit/receive antennas is separated differently). In other words, the BS simultaneously transmits and receives data over the same frequency, but the UE either transmits (uplink) or receives (downlink) data with the same radio resource. Recently, a dual-separated antenna architecture for IBFD radios has been proposed, which enables the BS to operate FD communication by isolating transmit and receive antenna panels [11,36]. It is also assumed that self-interference at the BS is completely canceled [13,31]. The BS wants to transmit a set of independent messages $\left(W_{1}^{d},\ldots ,W_{N_{d}}^{d}\right)$ to the downlink UE and receive a set of independent messages $\left(W_{1}^{u},\ldots ,W_{N_{u}}^{u}\right)$ from the uplink UE [14,35]. Because the BS operates in FD mode, the signals from uplink UE may interfere with downlink UE, which is called *cross-link interference (CLI)*.

Let $H_{i}^{d}\;\left(\in C^{L_{d}\times M}\right)$ be the channel matrix from the BS to the $i\;(\in N_{d}≜\left\{1,\ldots ,N_{d}\right\})$th downlink UE, $H_{j}^{u}\;\left(\in C^{M\times L_{u}}\right)$ be the channel matrix from the $j\;(\in N_{u}≜\left\{1,\ldots ,N_{u}\right\})$th uplink UE to the BS, and $H_{i,j}\;\left(\in C^{L_{d}\times L_{u}}\right)$ be the channel matrix from the *j*th uplink UE to the *i*th downlink UE. Each element of all channel matrices: $H_{i}^{d}$, $H_{j}^{u}$, and $H_{i,j}$, is assumed to follow an independent and identically distributed (i.i.d.) zero-mean and unit-variance circular symmetric complex Gaussian distribution, i.e., CN(0,1). In addition, block fading channels are assumed in this paper, i.e., the channel coefficients remain constant for at least one transmission block, but vary to independent values for different transmission blocks. The *i*th downlink UE is assumed to know its $H_{i}^{d}$ and the *j*th uplink UE is assumed to know its $H_{j}^{u}$ through the pilot signals broadcast from the BS and the channel reciprocity property [35]. Note that each node only has CSI with its index, called *local CSI*.

### 2.1. Transmission and Reception Signal Model

**Figure 1:**
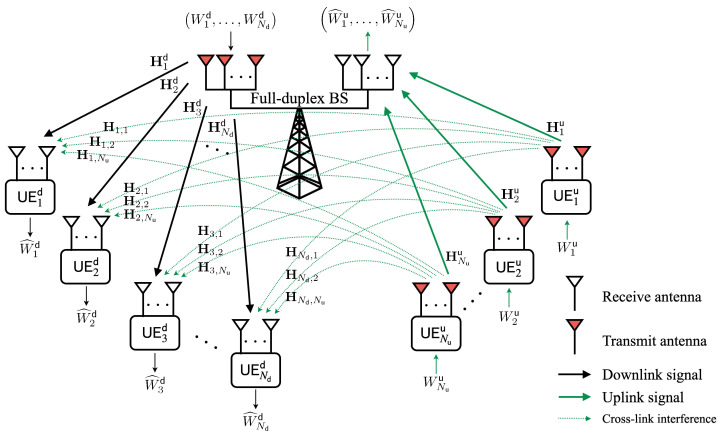
System model of an in-band full-duplex (IBFD) MIMO cellular network.

Figure 1 illustrates an example of the IBFD MIMO cellular network. The received signal vector at the *i*th downlink UE, denoted by $y_{i}^{d}\;\left(\in C^{L_{d}\times 1}\right)$, can be expressed as follows:

(1) $$y_{i}^{d}=H_{i}^{d}V_{\mathrm{BS}}^{d}x_{\mathrm{BS}}^{d}+\sum_{j=1}^{N_{u}}H_{i,j}v_{j}^{u}x_{j}^{u}+z_{i}^{d},\;$$

where $V_{\mathrm{BS}}^{d}\;\left(\in C^{M\times N_{d}}\right)$ and $x_{\mathrm{BS}}^{d}\;\left(\in C^{N_{d}\times 1}\right)$ represent the transmit beamforming (or precoding) matrix for decorrelating multiple spatial streams to downlink UE and the transmit signal vector of the BS, respectively, where $N_{d}\leq M$. The second term on the right-hand side represents the CLI, where $v_{j}^{u}\;\left(\in C^{L_{u}\times 1}\right)$ and $x_{j}^{u}\;\left(\in C\right)$ denote the transmit beamforming vector and the transmit signal of the *j*th uplink UE, respectively. We assume that each uplink UE sends a *single* data stream and each downlink UE also receives a *single* data stream; $z_{i}^{d}\;\left(\in C^{L_{d}\times 1}\right)$ is the additive white Gaussian noise (AWGN) at the *i*th downlink UE that follows an i.i.d. $\mathrm{CN}(0,N_{0}\cdot I_{L_{d}})$, where $N_{0}$ and $I_{L}$ denote the noise power and the L×L identity matrix, respectively, i.e., $z_{i}^{d}∼\mathrm{CN}\left(0,N_{0}\cdot I_{L_{d}}\right),\;\forall i\in N_{d}$. We also assume that the BS and all uplink UE satisfy the average transmission power constraint, such that $E[∥x_{\mathrm{BS}}^{d}{∥}^{2}]\leq P_{\mathrm{BS}}$ and $E[|x_{j}^{u}{|}^{2}]\leq P_{\mathrm{UE}},\;\forall j\in N_{u}$, respectively.

The *i*th downlink UE applies its receive beamforming vector defined as $u_{i}^{d}\;\left(\in C^{L_{d}\times 1}\right)$ to the received signal in (1) to recover its signal as follows:

(2) $${\tilde{y}}_{i}^{d}={\left(u_{i}^{d}\right)}^{T}\cdot y_{i}^{d}.\;$$

Meanwhile, the received signal vector at the BS, denoted by $y_{\mathrm{BS}}^{u}\;\left(\in C^{M\times 1}\right)$, can be written as follows:

(3) $$y_{\mathrm{BS}}^{u}=\sum_{j=1}^{N_{u}}H_{j}^{u}v_{j}^{u}x_{j}^{u}+z_{\mathrm{BS}}^{u}=\left[H_{1}^{u}v_{1}^{u},H_{2}^{u}v_{2}^{u},\ldots ,H_{N_{u}}^{u}\;v_{N_{u}}^{u}\right]{\left[x_{1}^{u},\ldots ,x_{N_{u}}^{u}\right]}^{T}+z_{\mathrm{BS}}^{u},$$

where $z_{\mathrm{BS}}^{d}\;\left(\in C^{M\times 1}\right)$ is the AWGN at the BS that conforms to an i.i.d. $\mathrm{CN}(0,N_{0}\cdot I_{M})$. The BS applies a receive beamforming (or post-coding) matrix defined as $U_{\mathrm{BS}}^{u}\;\left(\in C^{N_{u}\times M}\right)$ to this signal for decorrelating multiple spatial streams of uplink UE as follows:

(4) $${\tilde{y}}_{\mathrm{BS}}^{u}=U_{\mathrm{BS}}^{u}\cdot y_{\mathrm{BS}}^{u}.$$

We will present in Section 3 two design schemes for beamformers: $V_{\mathrm{BS}}^{d}$, $v_{j}^{u}$, $U_{\mathrm{BS}}^{u}$, and $u_{i}^{d}$ to attain the achievable sum-degrees-of-freedom (DoF) in the IBFD MIMO network.

### 2.2. Degrees-of-Freedom (DoF)

Let us define a set of length *n* block codes and its achievable DoF [14,35]. The DoF is a key performance metric for IA techniques and represents the signaling dimension, the number of interference-free AWGN channels equivalently, for a sufficiently high signal-to-noise ratio (SNR) [18]. Supposing that $W_{i}^{d}$ and $W_{j}^{u}$ are drawn from discrete uniform distributions $U\{1,2^{nR_{i}^{d}}\}$ and $U\{1,2^{nR_{j}^{u}}\}$, respectively, a code sequence $\left(2^{nR_{1}^{d}},\ldots ,2^{nR_{N_{d}}^{d}},2^{nR_{1}^{u}},\ldots ,2^{nR_{N_{u}}^{u}}\right)$ consists of the following set of encoding and decoding functions:

- *Encoding*: The encoding function of the BS is given by $x_{\mathrm{BS}}^{d}\left(l\right)=\Psi_{l}\left(W_{1}^{d},\ldots ,W_{N_{d}}^{d}\right)$, where l∈{1,…,n}. Similarly, the encoding function of the *j*th uplink UE is given by $x_{j}^{u}\left(l\right)=\phi_{l}\left(W_{j}^{u}\right)$.
- *Decoding*: Upon receiving signal vectors from $y_{\mathrm{BS}}^{u}\left(1\right)$ to $y_{\mathrm{BS}}^{u}\left(n\right)$, the decoding function of the BS is given by ${\hat{W}}_{j}^{u}=\Phi_{j}\left(y_{\mathrm{BS}}^{u}\left(1\right),\ldots ,y_{\mathrm{BS}}^{u}\left(n\right)\right)$. Similarly, upon receiving signal vectors from $y_{i}^{d}\left(1\right)$ to $y_{i}^{d}\left(n\right)$, the decoding function of the *i*th downlink UE is given by ${\hat{W}}_{i}^{d}=\psi_{i}\left(y_{i}^{d}\left(1\right),\ldots ,y_{i}^{d}\left(n\right)\right)$.

A tuple $\left(R_{1}^{d},\ldots ,R_{N_{d}}^{d},R_{1}^{u},\ldots ,R_{N_{u}}^{u}\right)$ is stated as *achievable rate* for the IBFD cellular network if there exists a code sequence of $\left(2^{nR_{1}^{d}},\ldots ,2^{nR_{N_{d}}^{d}},2^{nR_{1}^{u}},\ldots ,2^{nR_{N_{u}}^{u}}\right)$ such that $\mathrm{Pr}({\hat{W}}_{i}^{d}\neq W_{i}^{d})\to 0$ and $\mathrm{Pr}({\hat{W}}_{j}^{u}\neq W_{j}^{u})\to 0$ as *n* increases for all $i\in N_{d}$ and $j\in N_{u}$. Then, the achievable DoF tuple is given by

(5) $$\left(\eta_{1}^{d},\ldots ,\eta_{N_{d}}^{d},\eta_{1}^{u},\ldots ,\eta_{N_{u}}^{u}\right)=\lim_{\rho \to \infty }\left(\frac{R_{1}^{d}}{\log\rho },\ldots ,\frac{R_{N_{d}}^{d}}{\log\rho },\frac{R_{1}^{u}}{\log\rho },\ldots ,\frac{R_{N_{u}}^{u}}{\log\rho }\right),$$

where $\eta_{i}^{d}$ and $\eta_{j}^{u}$ denote the achievable DoF of the *i*th downlink UE and the *j*th uplink UE, respectively, and ρ represents the system SNR [18]. The DoFs of downlink and uplink are defined as

(6) $$\eta_{d}=\sum_{i=1}^{N_{d}}\eta_{i}^{d}\;\;\mathrm{and}\;\;\eta_{u}=\sum_{j=1}^{N_{u}}\eta_{j}^{u},$$

respectively. We then define the achievable sum-DoF for the IBFD MIMO network as follows:

(7) $$\eta_{\sum }=\eta_{d}+\eta_{u}.$$

## 3. QoS-Adaptive Interference Alignment for IBFD MIMO Network

We first summarize the main result of this paper in terms of the achievable sum-DoF and then elaborate on the overall procedure of the proposed IA schemes for achieving this DoF.

### 3.1. Main Result

Consider the IBFD MIMO network described in Section 2. Since we assume the number of candidate UE for data transmission or reception in both uplink and downlink, the DoF is not limited by $N_{d}$ or $N_{u}$. Practical cellular networks satisfy this assumption in general.

**Theorem** **1.** *The following DoF is achievable in the IBFD MIMO network via the proposed IA technique*

(8) $$\eta_{\sum }\;=\;\left\{\begin{array}{cc} 2M, & if\;\max\{L_{d},L_{u}\}>M,\\ M\;+\;\max\{L_{d},L_{u}\}\;-\;1, & if\;\max\{L_{d},L_{u}\}\leq M. \end{array}\right.$$

**Proof.**  We refer to Section 3.2 and Section 3.3 for the proof. □

**Remark** **1.** *The DoF of the conventional HD MIMO network is given by M, and thus the proposed technique achieves 100% DoF gain when $\max\{L_{d},L_{u}\}>M$. Furthermore, if we consider the symmetric IBFD MIMO cellular networks in which BS and all UE have the same number of antennas, M, then the achievable DoF of the proposed technique is given by 2M−1.*

### 3.2. Achievable Scheme 1: $L_{d}\geq L_{u}$

For the first achievable scheme, we assume that $L_{d}\geq L_{u}$, $N_{d}=M$, and $N_{u}=\min\left\{M,L_{d}-1\right\}$, where M≥1, $L_{d}\geq 2$, and $L_{u}\geq 1$. Under this assumption, it will be proved that the DoF in Theorem 1 is achievable via the proposed IA scheme, where each downlink UE aligns the CLI into the signal space of the other downlink UE. In particular, this IA scheme can also be derived from [35] as a special case; please see Appendix A for details. Note that all UE and the BS exploit their local CSI. This means that each node utilizes only the channels, including its index, to design the beamformer.

#### 3.2.1. Transmit Beamforming at Uplink UE

In the first achievable scheme, transmit beamforming vectors at the uplink UE are preferentially designed. By exploiting local CSI, the transmit beamforming vector of the *j*th uplink UE is designed by

(9) $$v_{j}^{u}=\arg\max_{{∥v∥}^{2}=1}{∥H_{j}^{u}\cdot v∥}^{2},$$

which maximizes the desired signal strength of the *j*th uplink UE at the BS. In fact, $v_{j}^{u}$ is the right-singular vector corresponding to the largest singular value of $H_{j}^{u}$. Note that such a transmit beamforming does not increase the DoF of the IBFD network even if it may increase the uplink sum rate.

Each uplink UE feeds its transmit beamforming vector back to the BS so that the BS decorrelates multiple uplink signals from the uplink UE. The BS then obtains effective channel vectors from the uplink UE to itself, denoted by

(10) $$g_{j}^{u}\;\left(\in C^{M\times 1}\right)≜H_{j}^{u}v_{j}^{u},\forall j\in N_{u}.$$

At this time, we assume that the *i*th downlink UE also obtains effective channel vectors between itself and the uplink UE, $H_{i,j}v_{j}^{u},\forall j\in N_{u}$, by overhearing the feedback signals of the uplink UE.

#### 3.2.2. Receive Beamforming at Downlink UE

Next, to cope with both the CLI from the uplink UE and the inter-stream interference (also called inter-user interference) from the BS, receive beamforming vectors are designed at the downlink UE; each is chosen as

(11) $$u_{i}^{d}=u\in \mathrm{Null}\left({\left[H_{i}^{\mathrm{CLI}}\right]}^{T}\right)$$

where $\mathrm{Null}(A^{T})$ ($=\{u|A^{T}u=0,∥u{∥}^{2}=1\}$) denotes the left null space of A and $H_{i}^{\mathrm{CLI}}$ ($≜\left[H_{i,1}v_{1}^{u},\ldots ,H_{i,N_{u}}\;v_{N_{u}}^{u}\right]\in C^{L_{d}\times N_{u}}$) denotes the equivalent CLI channel matrix from $N_{u}$ uplink UE to the *i*th downlink UE considering transmit beamforming vectors of the uplink UE. It is worth noting that such a vector $u_{i}^{d}$ in (11) always exists because $L_{d}>N_{u}$ by assumption.

Each downlink UE feeds its receive beamforming vector back to the BS for multiplexing multiple downlink signals at the BS. Then, the BS obtains effective channel vectors from itself to the downlink UE, denoted by

(12) $$g_{i}^{d}\;\left(\in C^{1\times M}\right)≜{\left(u_{i}^{d}\right)}^{T}H_{i}^{d},\forall i\in N_{d}.$$

#### 3.2.3. Pre- and Post-Coding at BS

The BS needs a pre-coding matrix for decorrelating multiple spatial streams to the downlink UE. Recall that we assume $N_{d}=M$ in the first achievable scheme. To cancel out inter-spatial interference among downlink UE, the pre-coding matrix $V_{\mathrm{BS}}^{d}$ is designed by exploiting effective channel vectors in (12) as follows:

(13) $$V_{\mathrm{BS}}^{d}=\left[v_{1}^{d},v_{2}^{d},\ldots ,v_{M}^{d}\right]={\left[\begin{array}{c} {\left(u_{1}^{d}\right)}^{T}H_{1}^{d}\\ {\left(u_{2}^{d}\right)}^{T}H_{2}^{d}\\ \vdots \\ {\left(u_{M}^{d}\right)}^{T}H_{M}^{d} \end{array}\right]}^{-1}\left[\begin{array}{cccc} \sqrt{\gamma_{1}} & 0 & \cdots & 0\\ 0 & \sqrt{\gamma_{2}} & \cdots & 0\\ \vdots & \vdots & \ddots & \vdots \\ 0 & 0 & \cdots & \sqrt{\gamma_{M}} \end{array}\right],$$

where $\sqrt{\gamma_{i}}\;(≜1/∥v_{i}^{d}∥)$ is a normalization factor for meeting the transmit power constraint for each spatial stream [26].

The BS also needs a post-coding matrix $U_{\mathrm{BS}}^{u}$ for decorrelating multiple spatial streams of uplink UE. By using effective channel vectors in (10), this is given by

(14) $$U_{\mathrm{BS}}^{u}={\left[g_{1}^{u},\ldots ,g_{N_{u}}^{u}\right]}^{†}={\left[H_{1}^{u}v_{1}^{u},\ldots ,H_{N_{u}}^{u}\;v_{N_{u}}^{u}\right]}^{†},$$

where $A^{†}$ denotes the pseudo-inverse of matrix A.

### 3.3. Achievable Scheme 2: $L_{d}\leq L_{u}$

For the second achievable scheme, we assume that $L_{d}\leq L_{u}$, $N_{d}=\min\left\{M,L_{u}-1\right\}$, and $N_{u}=M$, where M≥1, $L_{d}\geq 1$, and $L_{u}\geq 2$. Under this assumption, it will be proved that the DoF in Theorem 1 is achievable via the proposed IA scheme, where each uplink UE aligns the CLI into the null space of the downlink UE’s signal space. Note that all UE and the BS exploit their local CSI as well.

#### 3.3.1. Receive Beamforming at Downlink UE

In the second achievable scheme, receive beamforming vectors at the downlink UE are preferentially designed, while the transmit beamforming at the uplink UE is designed prior to the receive beamforming at the downlink UE in the first achievable scheme in Section 3.2. By exploiting local CSI, the receive beamforming vector of the *i*th downlink UE is designed by

(15) $$u_{i}^{d}=\arg\max_{{∥u∥}^{2}=1}{∥u^{H}\cdot H_{i}^{d}∥}^{2},$$

which maximizes the desired signal strength of the *i*th downlink UE. In fact, $u_{i}^{d}$ is the left-singular vector corresponding to the largest singular value of $H_{i}^{d}$. Note that such a receive beamforming does not increase the DoF of the IBFD network even if it may increase the downlink sum rate.

As in Section 3.2.1 and Section 3.2.2, each downlink UE feeds its receive beamforming vector back to the BS; then, the BS obtains $g_{i}^{d}$ in (12) and the *j*th uplink UE also obtains effective channel vectors between itself and the downlink UE, ${\left(u_{i}^{d}\right)}^{T}H_{i,j},\forall i\in N_{d}$, by overhearing the feedback signals of the downlink UE.

#### 3.3.2. Transmit Beamforming at Uplink UE

The transmit beamforming vector of the *j*th uplink UE is chosen as

(16) $$v_{j}^{u}=v\in \mathrm{Null}\left({\bar{H}}_{j}^{\mathrm{CLI}}\right)$$

where Null(A) ($=\{v|Av=0,∥v{∥}^{2}=1\}$) denotes the null space of A and ${\bar{H}}_{j}^{\mathrm{CLI}}$ ($≜{\left[{\left({\left(u_{1}^{d}\right)}^{T}H_{1,j}\right)}^{T},\ldots ,{\left({\left(u_{N_{d}}^{d}\right)}^{T}H_{N_{d},j}\right)}^{T}\right]}^{T}\in C^{N_{d}\times L_{u}}$) denotes the equivalent CLI channel matrix from the *j*th uplink UE to $N_{d}$ downlink UE considering receive beamforming vectors of the downlink UE. It is worth noting that such a vector $v_{j}^{u}$ in (16) always exists because $L_{u}>N_{d}$ by assumption.

As in Section 3.2.1, each uplink UE feeds its transmit beamforming vector back to the BS; then, the BS obtains effective channel vectors $g_{j}^{u}$ in (10). On the other hand, the BS can also obtain $g_{j}^{u}$ without feedback via pilot signals in the uplink streams.

#### 3.3.3. Pre- and Post-Coding at BS

The BS designs pre- and post-coding matrices, $V_{\mathrm{BS}}^{d}$ and $U_{\mathrm{BS}}^{u}$, with the same process in Section 3.2.3. Recall that we assume that $N_{d}=\min\left\{M,L_{u}-1\right\}$ in the second achievable scheme. Hence, both matrices in (13) and (14) are redesigned under this assumption as follows:

(17) $$V_{\mathrm{BS}}^{d}=\left[v_{1}^{d},v_{2}^{d},\ldots ,v_{N_{d}}^{d}\right]={\left[\begin{array}{c} {\left(u_{1}^{d}\right)}^{T}H_{1}^{d}\\ {\left(u_{2}^{d}\right)}^{T}H_{2}^{d}\\ \vdots \\ {\left(u_{N_{d}}^{d}\right)}^{T}H_{N_{d}}^{d} \end{array}\right]}^{†}\left[\begin{array}{cccc} \sqrt{\gamma_{1}} & 0 & \cdots & 0\\ 0 & \sqrt{\gamma_{2}} & \cdots & 0\\ \vdots & \vdots & \ddots & \vdots \\ 0 & 0 & \cdots & \sqrt{\gamma_{N_{d}}} \end{array}\right]$$

and

(18) $$U_{\mathrm{BS}}^{u}={\left[g_{1}^{u},\ldots ,g_{M}^{u}\right]}^{-1}={\left[H_{1}^{u}v_{1}^{u},\ldots ,H_{M}^{u}v_{M}^{u}\right]}^{-1},$$

respectively.

### 3.4. Data Transmission and Reception

Applying the receive beamforming in (11) for $L_{d}\geq L_{u}$ or (15) for $L_{d}\leq L_{u}$ at each downlink UE, the received signal (2) can be rewritten as follows:

(19) $$\begin{array}{cc} {\tilde{y}}_{i}^{d} & ={\left(u_{i}^{d}\right)}^{T}\cdot y_{i}^{d}\\ \; & ={\left(u_{i}^{d}\right)}^{T}\cdot H_{i}^{d}V_{\mathrm{BS}}^{d}x_{\mathrm{BS}}^{d}+{\left(u_{i}^{d}\right)}^{T}\cdot \sum_{j=1}^{N_{u}}H_{i,j}v_{j}^{u}x_{j}^{u}+{\left(u_{i}^{d}\right)}^{T}\cdot z_{i}^{d},\\ \; & =\sqrt{\gamma_{i}}x_{\mathrm{BS},i}^{d}+{\tilde{z}}_{i}^{d}, \end{array}\;$$

where $x_{\mathrm{BS},i}^{d}\;\left(\in C\right)$ denotes the transmit signal of the BS to the *i*th downlink UE and ${\tilde{z}}_{i}^{d}\;\left(\in C\right)$ is the noise after the receive beamforming; ${\tilde{z}}_{i}^{d}∼\mathrm{CN}\left(0,N_{0}\right)$ since $∥u_{i}^{d}{∥}^{2}=1$. It is worth noting that each downlink UE can achieve a single DoF; hence, the achievable downlink DoF becomes $N_{d}$.

After post-coding in (14) for $L_{d}\geq L_{u}$ and (18) for $L_{d}\leq L_{u}$ at the BS, the received signal (4) is given as follows:

(20) $$\begin{array}{cc} {\tilde{y}}_{\mathrm{BS}}^{u} & =U_{\mathrm{BS}}^{u}\cdot y_{\mathrm{BS}}^{u}\\ \; & =U_{\mathrm{BS}}^{u}\left[H_{1}^{u}v_{1}^{u},\ldots ,H_{N_{u}}^{u}\;v_{N_{u}}^{u}\right]{\left[x_{1}^{u},\ldots ,x_{N_{u}}^{u}\right]}^{T}+U_{\mathrm{BS}}^{u}\cdot z_{\mathrm{BS}}^{u},\\ \; & ={\left[x_{1}^{u},\ldots ,x_{N_{u}}^{u}\right]}^{T}+{\tilde{z}}_{\mathrm{BS}}^{u}, \end{array}\;$$

where ${\tilde{z}}_{\mathrm{BS}}^{u}\;\left(\in C^{N_{u}\times 1}\right)∼\mathrm{CN}\left(0,U_{\mathrm{BS}}^{u}\;\cdot \;{\left(U_{\mathrm{BS}}^{u}\right)}^{H}N_{0}\right)$. Note that the uplink signals for $N_{u}$ uplink UE are directly obtained from (20); hence, the achievable uplink DoF is equal to $N_{u}$.

### 3.5. Achievable Rate and DoF

We theoretically characterize the achievable rate and DoF of the proposed IA technique. From (19), the achievable rate of the *i*th downlink UE is given by

(21) $$R_{i}^{d}=\log_{2}\left(1+\frac{\gamma_{i}\cdot {\left|x_{\mathrm{BS},i}^{d}\right|}^{2}}{|{\tilde{z}}_{i}^{d}{|}^{2}}\right)=\log_{2}\left(1+\frac{\gamma_{i}\cdot \rho_{\mathrm{BS}}}{N_{d}}\right),$$

where $\rho_{\mathrm{BS}}\;(≜E[∥x_{\mathrm{BS}}^{d}{∥}^{2}]/E[|{\tilde{z}}_{i}^{d}{|}^{2}]=P_{\mathrm{BS}}/N_{0})$ denotes the transmit SNR of the BS. We assume that the BS evenly allocates transmit power to $N_{d}$ downlink UE, i.e., $E[|x_{\mathrm{BS},i}^{d}{|}^{2}]=P_{\mathrm{BS}}/N_{d}$, $\forall i\in N_{d}$. The sum rate of downlink UE is then expressed as

(22) $$R_{d}=\sum_{i=1}^{N_{d}}R_{i}^{d}.$$

Hence, the achievable DoF of the downlink is given from (6) by

(23) $$\eta_{d}=\sum_{i=1}^{N_{d}}\eta_{i}^{d}=N_{d},\;∵\eta_{i}^{d}=\lim_{\rho_{\mathrm{BS}}\to \infty }\frac{R_{i}^{d}}{\log\rho_{\mathrm{BS}}}=1.$$

In the first achievable scheme, we assume that $N_{d}=M$, so the downlink DoF is given by *M*, and in the second achievable scheme, we assume that $N_{d}=\min\left\{M,L_{u}-1\right\}$, hence the downlink DoF is given by $\min\{M,L_{u}-1\}$.

From (20), the achievable rate of the *j*th uplink UE is given by

(24) $$R_{j}^{u}=\log_{2}\left(1+\frac{|x_{j}^{u}{|}^{2}}{|{\tilde{z}}_{j}^{u}{|}^{2}}\right)=\log_{2}\left(1+\frac{\rho_{\mathrm{UE}}}{{\left[U_{\mathrm{BS}}^{u}\cdot {\left(U_{\mathrm{BS}}^{u}\right)}^{H}\right]}_{jj}}\right),$$

where ${\tilde{z}}_{j}^{u}$ denotes the *j*th element of ${\tilde{z}}_{\mathrm{BS}}^{u}$, $\rho_{\mathrm{UE}}\;(≜E[|x_{j}^{u}{|}^{2}]/E[|z_{j}^{u}{|}^{2}]=P_{\mathrm{UE}}/N_{0})$ denotes the transmit SNR of the uplink UE, and ${\left[A\right]}_{jj}$ denotes the (j,j)-th element of matrix A. The sum rate of uplink UE is then given by

(25) $$R_{u}=\sum_{j=1}^{N_{u}}R_{j}^{u}.$$

Hence, the achievable DoF of the uplink is given from (6) by

(26) $$\eta_{u}=\sum_{j=1}^{N_{u}}\eta_{j}^{u}=N_{u},\;∵\eta_{j}^{u}=\lim_{\rho_{\mathrm{UE}}\to \infty }\frac{R_{j}^{u}}{\log\rho_{\mathrm{UE}}}=1,$$

where $N_{u}=\min\left\{M,L_{d}-1\right\}$ in the first achievable scheme and $N_{u}=M$ in the second achievable scheme.

Finally, the achievable sum-DoF of the proposed IA technique in the IBFD MIMO cellular network is given by

(27) $$\eta_{\sum }=\eta_{d}+\eta_{u}=N_{d}+N_{u}=\left\{\begin{array}{cc} M+\min\{M,L_{d}-1\}, & \;\mathrm{if}\;L_{d}\geq L_{u},\\ \min\{M,L_{u}-1\}+M, & \;\mathrm{if}\;L_{d}\leq L_{u}. \end{array}\right.$$

For the first achievable scheme ($L_{d}\geq L_{u}$), $\eta_{\sum }=2M$ if $L_{d}>M$, otherwise $\eta_{\sum }=M+L_{d}-1$; for the second achievable scheme ($L_{d}\leq L_{u}$), $\eta_{\sum }=2M$ if $L_{u}>M$, otherwise $\eta_{\sum }=M+L_{u}-1$. This proves the Theorem 1.

## 4. Simulation Results

We show the achievable sum rate performance of the proposed IA technique through Monte Carlo simulations. Note that the sum rate is a suitable metric for measuring the underlying performance of IA techniques than DoF, which reflects macroscopic behavior [18]. To the best of our knowledge, since there is no distributed IA scheme for IBFD MIMO networks, as in [33,35], we compare the proposed IA technique with the HD case in which the BS operates in HD mode, which can be regarded as multi-user MIMO (MU-MIMO), and the ideal case in which there exists no CLI. Although there is an IA technique in [13] for the IBFD MIMO network based on the minimum mean squared error (MMSE) method with global CSI, this is not applicable in our IBFD MIMO networks because each node has only local CSI. In particular, CSI exchange between all nodes for global CSI results in significant signaling overhead, which is infeasible in practical networks. Moreover, for a fair comparison, it is necessary to elaborate on the rate loss due to CSI sharing, but it is beyond the scope of this paper. The sum rate performance in the IBFD MIMO network without CLI represents the upper bound of the proposed IA technique. We consider two IBFD MIMO network scenarios: the number of BS antennas 2M=6, the number of antennas at each downlink UE $L_{d}=3$, the number of antennas at each uplink UE $L_{u}=2$, the number of downlink UE $N_{d}=3$, the number of uplink UE $N_{u}=2$ for the first achievable scheme; and M=3, $L_{d}=2$, $L_{u}=3$, $N_{d}=2$, $N_{u}=3$ for the second achievable scheme. We also assume that transmission power per UE is the same for uplink and downlink, i.e., $P_{\mathrm{BS}}=N_{d}P_{\mathrm{UE}}$.

**Figure 2:**
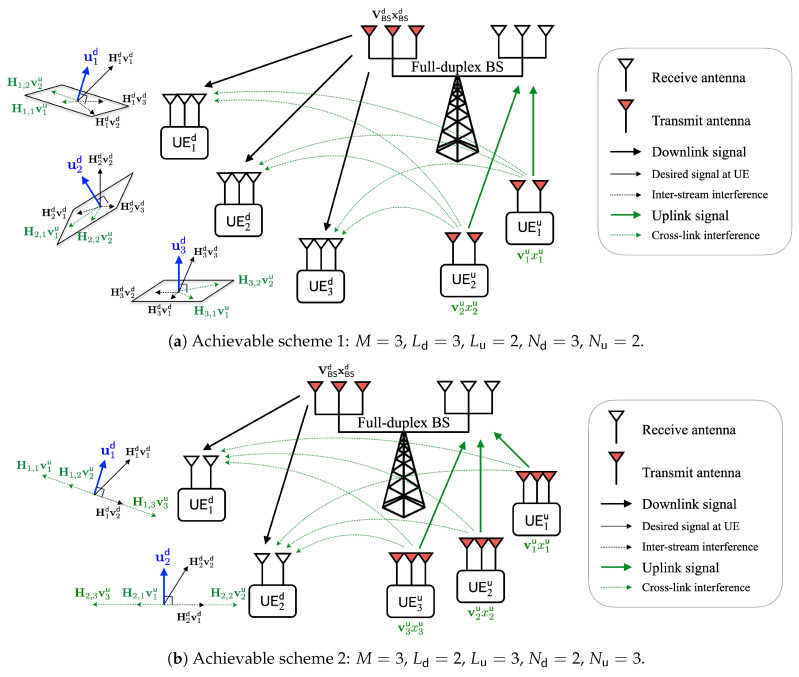
Geometric interpretation of the proposed IA schemes in IBFD MIMO networks.

Figure 2 shows the geometric interpretation of the proposed IA schemes in Section 3.2 and Section 3.3 to illustrate the simulation scenarios. In particular, Figure 2a,b depict the geometrical aspects of the received signals at the downlink UE for achievable schemes 1 and 2, respectively. In both figures, the number of BS antennas is set to 6, i.e., M=3. For a certain downlink UE, the signals to the other downlink UE (inter-stream interference) are *aligned* into the space spanned by the CLI. For example, at the third downlink UE (denoted as `${\mathrm{UE}}_{3}^{d}$’ in Figure 2a), four interferences (two CLIs and two inter-stream interferences) are confined into two-dimensional space orthogonal to the receive beamforming vector $u_{3}^{d}$. In Figure 2b, at the third downlink UE, four interferences (three CLIs and one inter-stream interference) are confined into one-dimensional space orthogonal to the receive beamforming vector $u_{3}^{d}$.

**Figure 3:**
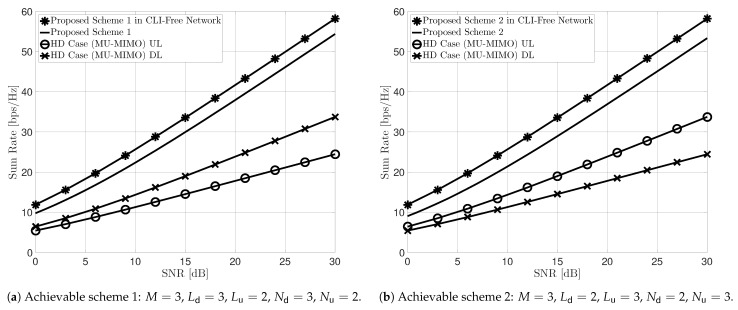
Sum rate performance of the proposed IA schemes, ideal IBFD MIMO (without CLI), and HD case (MU-MIMO) for uplink and downlink.

**Figure 4:**
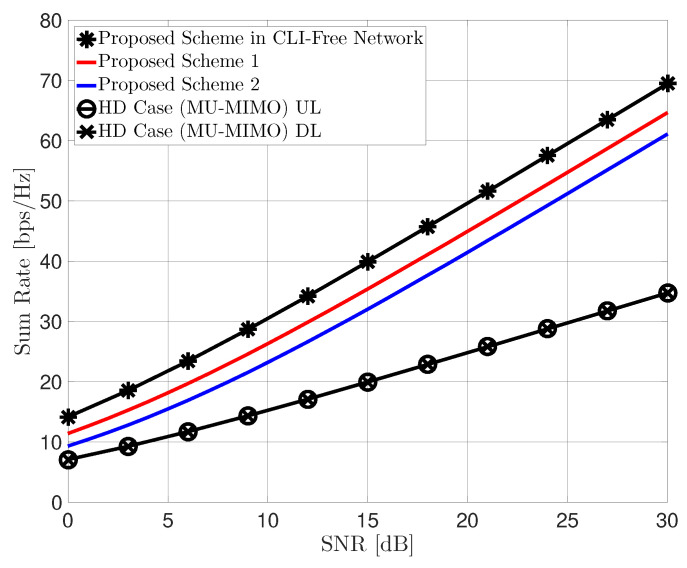
Sum rate performance of the proposed IA schemes: M=3, $L_{d}=4$, $L_{u}=4$, $N_{d}=3$, $N_{u}=3$.

Figure 3 shows the achievable sum rate with respect to the SNR of the proposed IA technique and compares it with the aforementioned benchmarks. In particular, Figure 3a,b show the sum rate performance of the achievable schemes 1 and 2, respectively. In both figures, the achievable sum-DoF of the proposed IA schemes is equal to 5, while the HD case (MU-MIMO) achieves a DoF of 3 or 2. More specifically, the achievable DoF of the HD case is 3 for downlink and 2 for uplink in scenario 1, and 2 for downlink and 3 for uplink in scenario 2, respectively. We can observe that the FD communication at the BS significantly outperforms the HD mode. Moreover, it is shown that the proposed two IA schemes have comparable performance to ideal cases and achieve the same slope, which means that the proposed IA technique can achieve the same DoF as in the CLI-free IBFD MIMO networks. Finally, we further consider a symmetric scenario with the same number of uplink and downlink UE: M=3, $L_{d}=4$, $L_{u}=4$, $N_{d}=3$, $N_{u}=3$ as shown in Figure 4. In this scenario, the achievable DoF of the proposed IA schemes is 6, while the HD case achieves a DoF of 3. Note that for the symmetric scenario $L_{d}=L_{u}$, both proposed IA schemes are available. We can observe from this figure that achievable scheme 1 slightly outperforms 2 when there are the same number of UE for uplink and downlink with the same number of antennas. Nevertheless, the achievable scheme 2 is still meaningful in that it can omit the feedback procedure of the uplink UE.

## 5. Conclusions

In-band full-duplex (IBFD) communication is one of the promising technologies for next-generation cellular networks. In this paper, we have investigated a practical IBFD MIMO cellular network in which a BS operates full-duplex (FD) communication and user equipment (UE) communicates in half-duplex (HD) mode. These IBFD networks introduce a new interference source called cross-link interference (CLI). We proposed a novel distributed interference alignment (IA) technique to alleviate the CLI. Briefly, the proposed IA technique consists of two achievable schemes: the *first* achievable scheme is that each downlink UE aligns CLI into the other downlink UE’s signal space, and the *second* achievable scheme is that each uplink UE aligns CLI into the null space of the downlink UE’s signal space. Both schemes are symmetric and can be flexibly applied depending on the network QoS requirements, such as uplink and downlink traffic needs. Moreover, we have characterized and proved the achievable sum-DoF of the proposed IA technique. Simulation results have shown that the proposed IA technique significantly improves the sum rate performance compared to conventional HD communications while achieving the same achievable DoF as the CLI-free IBFD MIMO network. As a further study, we will mathematically analyze the sum rate performance of the proposed IA technique and investigate the optimality of the network parameters. 

## Data Availability

Not applicable.

## Abbreviations

The following abbreviations are used in this manuscript:

AWGN	Additive white Gaussian noise
BS	Base station
CLI	Cross-link interference
CSI	Channel state information
DL	Downlink
DoF	Degrees-of-freedom
FD	Full-duplex
HD	Half-duplex
IA	Interference alignment
i.i.d.	independent and identically distributed
IBFD	In-band full-duplex
MIMO	Multiple-input multiple-output
QoS	Quality-of-service
UE	User equipment
UL	Uplink

## Appendix A

Although the authors of [35] have dealt with a two-cell MIMO network with dynamic time-division duplex mode, not a single-cell IBFD MIMO network, their achievable scheme can be exploited for our system model. As a particular case of [35], we can consider a case without interference between the two BSs, i.e., $H_{\mathrm{BS}}=0_{M,M}$ in ([35] (1)), where $0_{M,M}$ denotes M×M zero matrix. This means that the rank of the channel matrix between two BSs is zero; hence, $U_{\mathrm{Null}}^{u}$ in ([35] (5)) can be given by $I_{M}$. Their IA technique then becomes the same as our first achievable scheme for $L_{d}=L_{u}$.
